# Supplementary material for: The establishment of prostate-specific, SKP2 humanized mice by CRISPR knock-in method reveals neoplastic initiation and microenvironmental reprogramming
Source: Oncogene. 2026 Aug 1;45(37):3984–96. doi: 10.1038/s41388-026-03905-7 (PMC13549951; doi:10.1038/s41388-026-03905-7)
Supplement: Supplementary file 1 [file 41388_2026_3905_MOESM1_ESM.pdf]

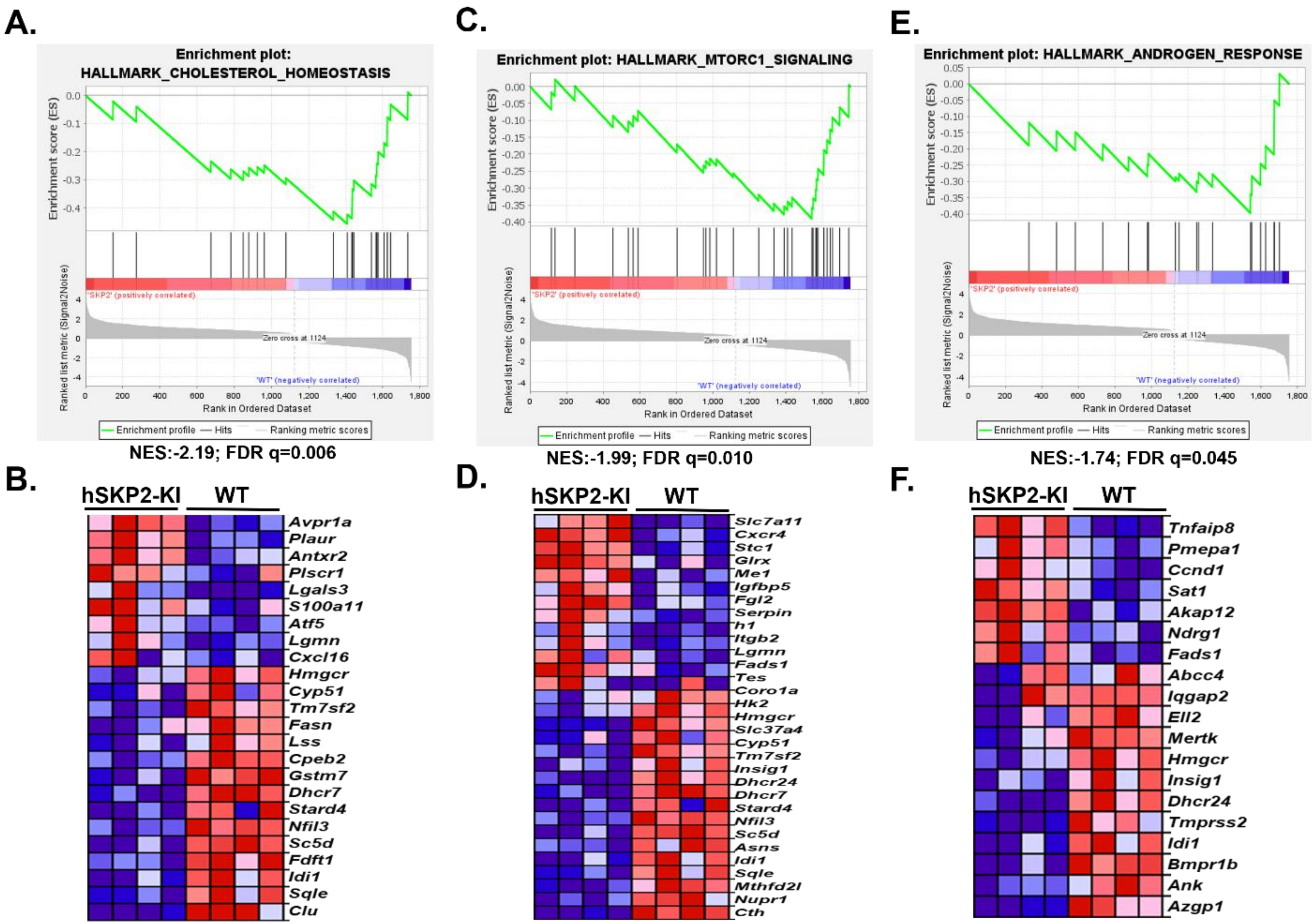

Supplementary Fig. 1. Highly enriched pathways contributed by significantly downregulated genes in h*SKP2*-KI mouse prostates. A. and B. GSEA enrichment plot of the cholesterol homeostasis pathway and the heatmap of core enriched genes. **C. and D.** GSEA enrichment plot of the mTORC1 pathway and the heatmap of core enriched genes. **E. and F.** GSEA enrichment plot of the androgen response pathway and the heatmap of core enriched genes.

Interferon responses

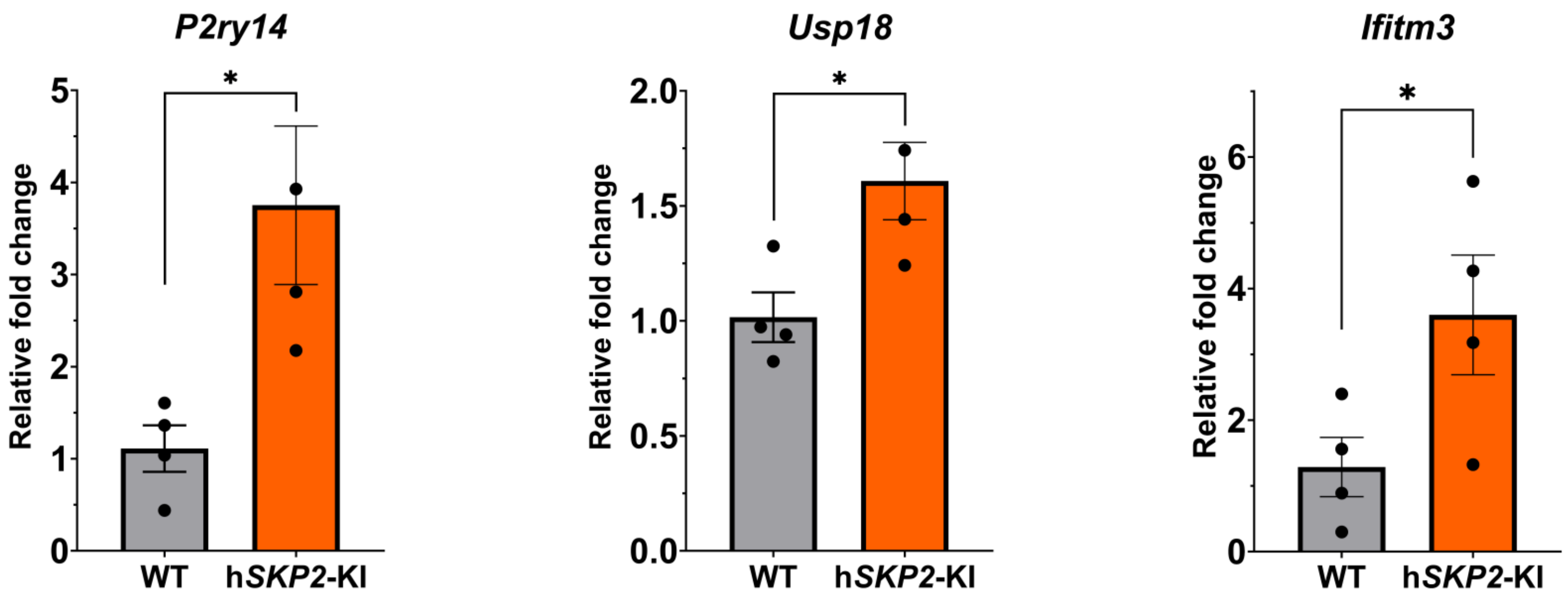

Supplementary Fig. 2. Quantitative PCR analysis of the expression of interferon-related genes *P2ry14*, *Usp18* and *Ifitm3* in the prostate of h*SKP2*-KI mice. N = 4. "*" and "**" denote $P < 0.05$ and < 0.01, respectively.

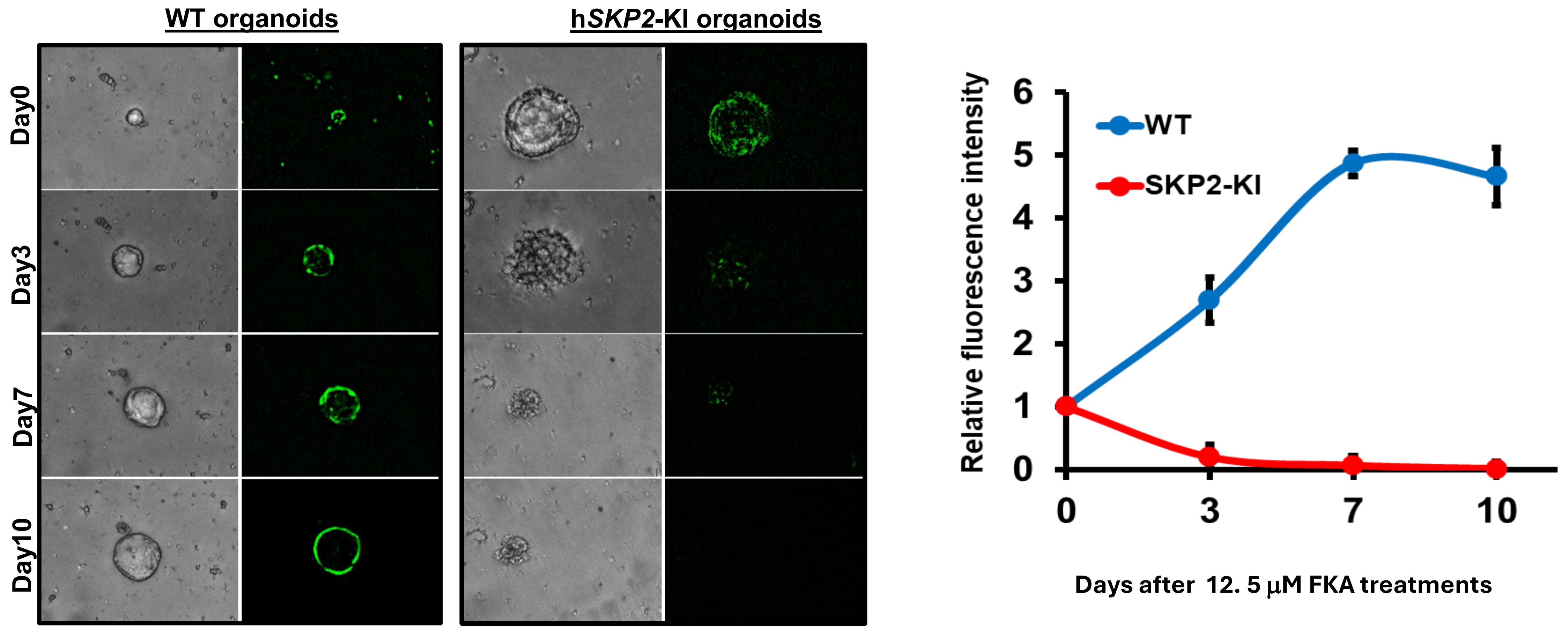

Supplementary Fig. 3. A. WT and h*SKP2*-KI mouse prostate organoids were treated with FKA at 12.5μM for 10 days. Viabilities of these organoids were examined by adding a fluorescent dye CalAM 30 minutes before fluorescent intensity reading and imaging at indicated time points, Scale bar: 100 μm. **B**. Relative fluorescent intensities of these organoids to their baseline levels at day 0 before treatments were calculated at indicated time points and presented as a line graph. ($Ps < 0.001$, One-way ANOVA test).

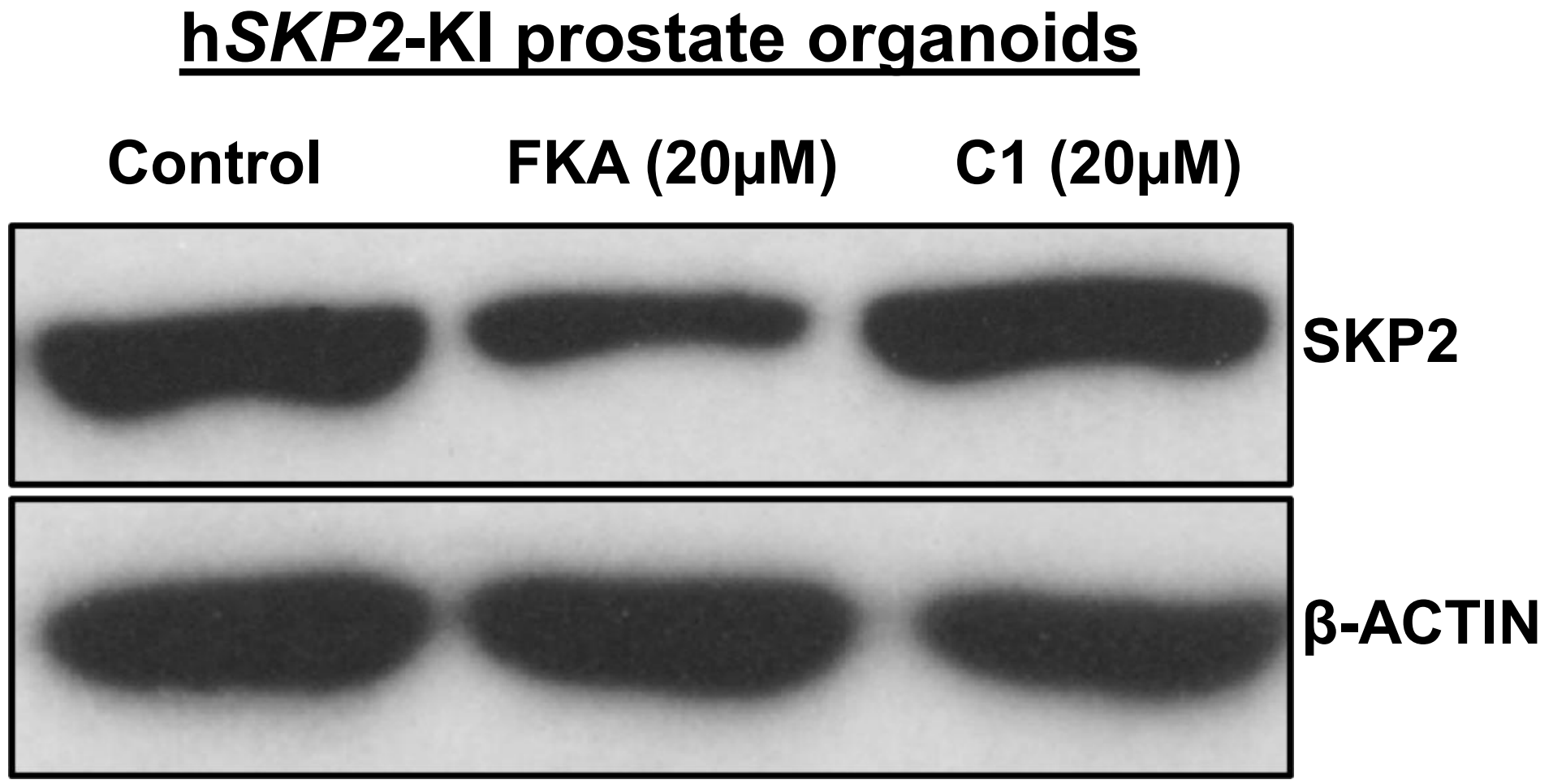

Supplementary Fig. 4. The effect of FKA and C1 on protein expression of SKP2 in hSKP2-KI mouse prostate organoids. Western blotting analysis of SKP2 protein expression after treatment of hSKP2-KI mouse prostate organoids with FKA and C1 for 5 days at a concentration of 20μM.

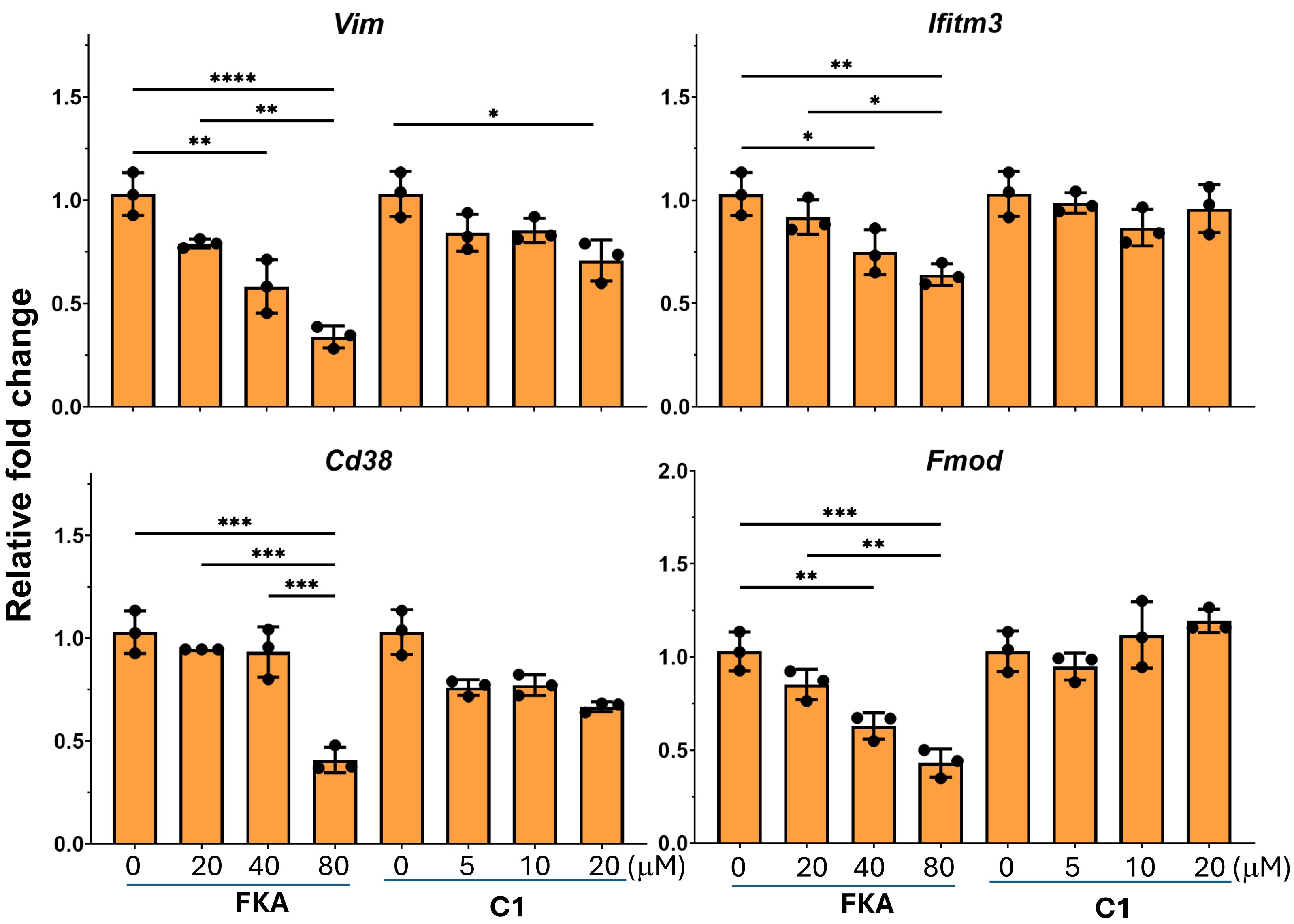

Supplementary Fig. 5. The effect of FKA and C1 on mRNA expression of EMT and interferon signaling related genes in hSKP2-KI mouse prostate organoids. *SKP2*-KI mouse prostate organoids were treated with FKA or C1 at indicated concentrations for 5 days and mRNA levels of *Vim*, *Ifitm3, Cd38,* and *Fmod* are examined. One-way ANOVA was conducted and post-ad hoc comparisons using the Bonferroni test. Data presented as mean + SD, n=3. Statistical significance is shown as *P < 0.05, **P < 0.01, ***P < 0.001, or ****P < 0.0001.
