# Supplementary material for: The establishment of prostate-specific, SKP2 humanized mice by CRISPR knock-in method reveals neoplastic initiation and microenvironmental reprogramming
Source: Oncogene. 2026 Aug 1;45(37):3984–96. doi: 10.1038/s41388-026-03905-7 (PMC13549951; doi:10.1038/s41388-026-03905-7)
Supplement: Supplementary file 2 [file 41388_2026_3905_MOESM2_ESM.docx]

**Supplementary Table 1:** Quantitative PCR primers

| **Gene** | **Species** | **Forward sequence (5'-3')** | **Reverse sequence (5'-3')** |
| --- | --- | --- | --- |
| *Fmod* | mouse | AGCAGTCCACCTACTACGACC | CAGTCGCATTCTTGGGGACA |
| *Thy1* | mouse | TGCTCTCAGTCTTGCAGGTG | TGGATGGAGTTATCCTTGGTGTT |
| *Wnt5a* | mouse | CAACTGGCAGGACTTTCTCAA | CATCTCCGATGCCGGAACT |
| *Cd38* | mouse | GGTCCAAGTGATGCTCAATGGG | AGCTCCTTCGATGTCGTGCATC |
| *Usp18* | mouse | GGAACCTGACTAAGGACCAGATC | GAGAGTGTGAGCAGTTTGCTCC |
| *Il15* | mouse | GTAGGTCTCCCTAAAACAGAGGC | TCCAGGAGAAAGCAGTTCATTGC |
| *Pfkp* | mouse | AAGAGGAAACCAAGCAGTGCGC | TTCCTCGGAGTTTCACGGCTTC |
| *Lats2* | mouse | GCACTGGATTCAGGTGGACTCA | CGACAGTTGGAAACATCGTCCC |
| *P2ry14* | mouse | CGACAGTTGGAAACATCGTCCC | GCTGTAGTGACCTTCCGTCTGA |
| *Tnfaip2* | mouse | TGTGCACCTGCACCTAGTGAA | CACTGGAATCTTGCAGGCGAA |
| *Gapdh* | mouse | CTCCCACTCTTCCACCTT CG | GCCTCTCTTGCTCAGTGTCC |
| *Ifitm3* | mouse | CCCCCAAACTACGAAAGAATCA | ACCATCTTCCGATCCCTAGAC |
| *SKP2* | human | CTGAGCTGCTAAAGGTCTCTGGTG | CACCCCTTGAGACAGCAACCGAC |
| *FMOD* | human | ATTGGTGGTTCCACTACCTCC | GGTAAGGCTCGTAGGTCTCATA |
| *THY1* | human | ATGAAGGTCCTCTACTTATCCGC | GCACTGTGACGTTCTGGGA |
| *WNT5a* | human | GCCAGTATCAATTCCGACATCG | TCACCGCGTATGTGAAGGC |
| *IFITM3* | human | CTGGGCTTCATAGCATTCGCCT | AGATGTTCAGGCACTTGGCGGT |
| *ACTB* | human | CACCATTGGCAATGAGCGGTTC | AGGTCTTTGCGGATGTCCACGT |
| *TNFAIP2* | human | TGCTCCAGAACCTGCATGAGGA | AACTCAGGCAGCCTCGTGTCTA |
| *USP18* | human | TGGACAGACCTGCTGCCTTAAC | CTGTCCTGCATCTTCTCCAGCA |
| *IL15* | human | AACAGAAGCCAACTGGGTGAATG | CTCCAAGAGAAAGCACTTCATTGC |
| *PFKP* | human | AGGCAGTCATCGCCTTGCTAGA | ATCGCCTTCTGCACATCCTGAG |
| *LATS2* | human | GTTCTTCATGGAGCAGCACGTG | CTGGTAGAGGATCTTCCGCATC |
| *P2RY14* | human | GCCGCAACATATTCAGCATCGTG | GCTGTAATGAGCTTCGGTCTGAC |
